# Universal fluctuations in growth dynamics of economic systems

**DOI:** 10.1038/s41598-018-38088-z

**Published:** 2019-01-24

**Authors:** Nathan C. Frey, Sakib Matin, H. Eugene Stanley, Michael A. Salinger

**Affiliations:** 1 Department of Physics, Boston University, Boston, MA 02215 USA; 2 Center for Polymer Studies, Boston University, Boston, MA 02215 USA; 3 Department of Markets, Public Policy and Law, Questrom School of Business, Boston University, Boston, MA 02215 USA

## Abstract

The growth of business firms is an example of a system of complex interacting units that resembles complex interacting systems in nature such as earthquakes. Remarkably, work in econophysics has provided evidence that the statistical properties of the growth of business firms follow the same sorts of power laws that characterize physical systems near their critical points. Given how economies change over time, whether these statistical properties are persistent, robust, and universal like those of physical systems remains an open question. Here, we show that the scaling properties of firm growth previously demonstrated for publicly-traded U.S. manufacturing firms from 1974 to 1993 apply to the same sorts of firms from 1993 to 2015, to firms in other broad sectors (such as materials), and to firms in new sectors (such as Internet services). We measure virtually the same scaling exponent for manufacturing for the 1993 to 2015 period as for the 1974 to 1993 period and virtually the same scaling exponent for other sectors as for manufacturing. Furthermore, we show that fluctuations of the growth rate for new industries self-organize into a power law over relatively short time scales.

## Introduction

Recently, the pursuit of statistical regularities in economics data and theoretical explanations have received increasing interest from both the physics and economics communities^1,2^. Using data on US manufacturing firms from 1974 to 1993, Stanley *et al*.^3,4^ documented that the standard deviations of the growth rates obey a power law with a scaling exponent of approximately −1/5^4–7^ and that the distribution of growth rates conditional on initial size is exponential over seven orders of magnitude^3,4^. These results resemble the power laws that are robust statistical properties of many complex interacting physical systems^2,8–11^. The theoretical explanation for such findings remains unclear. Models of critical phenomena in systems of strongly interacting elements predict results like those that have been found for firm growth, but so do models of weakly or non-interacting units^2,12–15^. Yet, to the extent that the above-mentioned results about the statistical properties of firm growth are stable, distinguishing among the competing explanations could provide important insights into the fundamental economic question of the nature of business firms^16^. On the other hand, if changes in economic conditions cause the statistical properties of firm growth to change significantly, then the need for a theoretical explanation is less compelling. Therefore, a natural question to ask about empirical relationships in economics is whether they are as stable as power laws in physical systems or, alternatively, whether they change or even fall apart as the economy changes.

## Results

**Figure 1:**
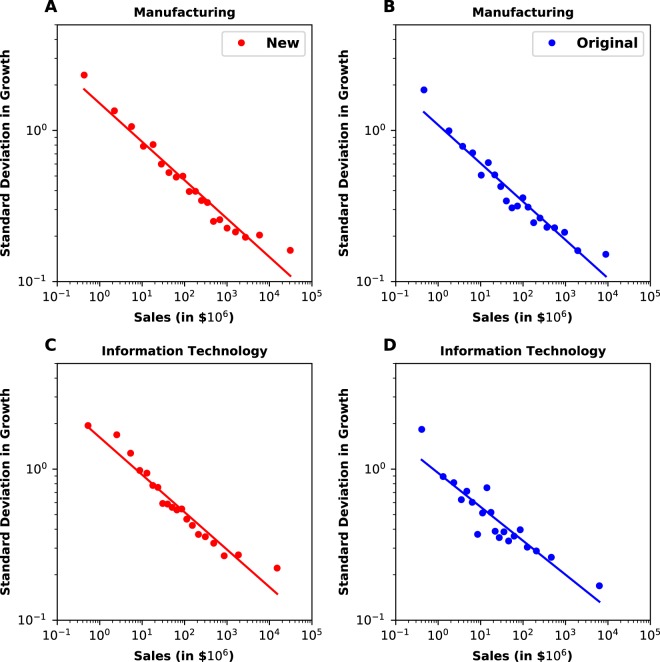
Scaling of fluctuations against growth for ‘Manufacturing’ and ‘Information Technology’ in ‘Original’ and ‘New’ time periods. The stability of the exponent over all time is strong evidence of universality.

Figure 1a,b are log-log plots of the standard deviation of one-year growth rates as a function of initial firm size for U.S. manufacturing firms over two time periods. Firm growth rate is defined as *R* = *S*_1_/*S*_0_, where *S*_1_ and *S*_0_ are consecutive annual measures of firm size. The two time periods are 1974–1993 (‘Original’), which is the period Stanley *et al*. analyzed, and 1993–2015 (‘New’). The standard deviation, *σ*(*S*_0_), is fit to a power law of the form $\sigma ({S}_{0})=a{S}_{0}^{-\beta }$, where *a* is a constant and *β* is the scaling exponent. For the Original time period *β* = 0.25 ± 0.02. For the New time period *β* = 0.26 ± 0.01. They are virtually identical to each other, and the results for the Original time period are not statistically significantly different from the 0.20 ± 0.03 reported by Stanley *et al*. for the same time period^4^. This agreement suggests that the power law, initially proposed and verified in 1996^3^, is quite robust. The linearity of this scaling and all other measured power law scaling was further tested using maximum likelihood estimation^15,17–19^ (see Supplementary Information and Methods Section).

We then extend our analysis to the ten other sectors (as categorized under the Global Industrial Classification System). For eight of the ten in the 1974–1993 datasets, we find a power law with the same (within error bars) scaling exponent as we measured for manufacturing. For example, we find $\beta =0.25\pm \mathrm{0.02,}\,0.22\pm \mathrm{0.02,}\,0.25\pm 0.02$, and 0.22 ± 0.02 for the health care, industrial, materials, and information technology (Fig. 1d) sectors, respectively. Similarly, for more recent data from 1993-present, we find $\beta =0.25\pm \mathrm{0.01,}\,0.25\pm \mathrm{0.01,}\,0.23\pm 0.02$, and 0.25 ± 0.01 for the health care, industrial, materials, and information technology (Fig. 1c) sectors, respectively. Not only do fluctuations of the firm growth for each of these sectors obey a well-defined power law, the scaling exponents are approximately the same value for each sector. Plots for other sectors can be found in Fig. S1. Scaling exponents for all sectors over the Original and New time periods are reported in Tables S1 and S2, respectively. Interestingly, the utility sector does not follow a power law in either period; the telecommunications sector does not follow a power law in the earlier period; and the scaling exponent for the financial sector was not stable between the two periods as it dropped significantly in absolute value from the earlier to the later period. Why these sectors exhibit different growth dynamics remains a topic for future research.

An interesting question to consider is whether the growth dynamics of new sectors in the economy (such as biotechnology) have the same properties as those of more established sectors. If the statistical properties of firm growth in fact are an example of self-organized criticality, we might expect the scaling properties associated with critical phenomenon to emerge over time. To test this hypothesis, we identified three industries for which we have data from their inception: ‘Biotechnology’, ‘Software’, and ‘Internet Software and Services.’ We computed the standard deviation of logarithmic growth rates conditional on initial size for moving 5-year windows. For each 5-year window, we regressed the logarithm of the standard deviation on the logarithm of initial size.

**Figure 2:**
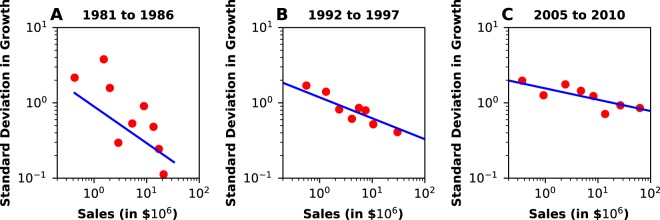
Scaling of Biotechnology Industry over 3 distinct time periods showing self-organization of a power law.

Figure 2 shows the results for the ‘Biotechnology’ sector. In Fig. 2a, which shows results for the window from 1981 to 1986, the standard error (SE) is 0.2 and it is clear that there is no power law relationship between the data. In contrast, over the time periods 1992–1997 (Fig. 2b) and 2005–2010 (Fig. 2c), a power law trend clearly emerges. The regression SE reduces to 0.02, and the scaling law accurately describes standard deviation in growth over 4 orders of magnitude. At the “birth” of a sector, few firms exist and there is no evidence of organization. Over the time period underlying Fig. 2a, there were only 53 publicly traded American biotechnology firms included in the analysis. The number increases to 214 (Fig. 2b) and 514 (Fig. 2c). Plots showing similar self-organization behavior are shown for the recently established Software and Internet Software & Services industries in Figs S2 and S3, respectively. Future work may probe and explain the dynamics of this self-organization behavior.

**Figure 3:**
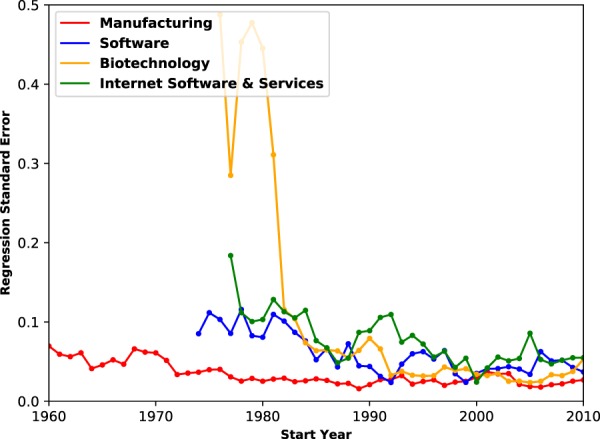
Times series of the regression standard error for different industries showing fast self-organization of power laws.

We extend the above analysis by considering time series of regression standard errors for pooled data for a variety of sectors, including biotechnology. As shown in Fig. 3 (red), the standard errors for the manufacturing sector regressions are fairly low, indicating good fit to a power law. Furthermore, we see there is a near-monotonic decrease of the SE. In contrast, the time series of the SE for all three new industries (‘Biotechnology’, ‘Software’, and ‘Internet Software and Services’) show a sharp decrease over some characteristic time scale. The SE for Biotechnology in 1980 is 0.5 and decreases to below 0.1 from 1984 to the present year. Similar trends are observed if we use the R-squared goodness of fit metric instead of SE. From this straightforward analysis, we conclude that there is a “convergence” towards power law behavior which is universal across different types of companies. Possible “convergence criteria” and models have been proposed^13–16,20–22^, which can be tested in future work.

## Conclusions

The results presented above enhance our understanding of the empirical facts that describe the dynamics of firm size and growth. The robustness of the observed scaling laws across different sectors over many orders of magnitude provides compelling evidence that general dynamical principles, not specific to particular industries, govern the growth of firms. The analysis of ‘new industries’ illuminates growth dynamics at the early stages. The self-organization of the scaling behavior for new industries provides new, stronger evidence of universality in economic systems.

## Methods

Data was collected from the Compustat database, which is available through Wharton Research Data Services. Compustat contains financial data on all firms that are publicly-traded on United States stock exchanges.

Companies were sorted according to the Global Industry Classification Standard (GICS). At the highest level, the economy is broken into “sectors”. In specific cases, we show power law behavior from the sector level, down through the “industry group” and “industry” divisions.

Several metrics for firm size can be used, such as sales, employees, etc. In the paper, we used the ‘net sales’ or revenue as the firm size, as is standard in the economic literature. We have conducted a similar analysis for employees and assets and the results are qualitatively similar to that for sales. These results are summarized for the manufacturing sector in Fig. S4.

We analyzed the standard deviation of growth rates, *R*, versus initial size, *S*_0_. The log-log scale is chosen such that a straight-line plot on the log-log plot corresponds to a power law, and the slope of the line corresponds to the scaling exponent. We extract the exponent by running a regression on a log-log scale. For each plot, we pooled all the firms’ initial sizes and yearly growth rates within a given industry or sector and over a given period of time. To compute the standard deviations for bins of growth rates, we used 20 bins when data is plotted at a sector level over the ‘Original’ and ‘New’ time periods. The ‘Original’ time corresponds to the same years as Stanley *et al*.^3^, whereas the ‘New’ time corresponds to all subsequent years. When analyzing new industries, we used 10 bins. We computed scaling exponents for 10, 20, and 30 bins for the manufacturing sector to verify that the computed exponents do not depend on the number of bins. These plots are shown in Fig. S4 and the scaling exponents are reported in Table S3. One-year growth rates of greater than 1000% were discarded as outliers.

The graphical method described above was used to determine the statistical structure of the growth residuals without needing to assume the underlying distribution of the data. Using this method, we determined the power law scaling for fluctuations of the growth rates. As in Canning *et al*.^17^, we performed a check on the exponents calculated via the graphical approach by assuming a power law distribution for the standard deviations of the growth residuals with the functional form log *σ*(*S*_0_) = log *a*–*β* log *S*_0_ + *γ*(log *S*_0_)^2^ and employed maximum likelihood estimation (MLE) to calculate the scaling exponents^15,17–19^. We set *γ* = 0 to explicitly test the log linearity of the scaling relations. Fits showing the overlap between the graphical method and the MLE method are shown for the ‘Information Technology’, ‘Manufacturing’, ‘Financials’, and ‘Materials’ sectors over the ‘Original’ and ‘New’ time periods in Fig. S5. MLE scaling exponents and standard errors are reported for all sectors over the ‘Original’ and ‘New’ time periods in Tables S1 and S2, respectively. For each sector, both MLE and ordinary least squares methods yield the same scaling exponent within error, further confirming the log linearity (power law) of the growth residuals over many orders of magnitude. We then performed a standard log-likelihood ratio test, as done in Canning *et al*.^17^, where the null hypothesis is that *γ* = 0. For all sectors over the Original time period, we cannot reject the null hypothesis at the 95% confidence interval, meaning that the linear model is a better fit for the scaling functions than the nonlinear model. We also fail to reject the null hypothesis for all sectors over the New time period, except Consumer Discretionary. For this sector, the presence of outlier observations at the tails of the distribution may mean that linearity only holds over a limited range. The *γ* values from MLE and p-values from hypothesis testing are reported for Original time in Table S4 and for New time in Table S5.

Stanley *et al*. analyzed the manufacturing industry between 1974 to 1993. The original data set was classified using the Standard Industrial Classification (SIC) method. The data available to us now follows the GICS classification. As such, the “original data” was constructed by pooling together appropriate industries to reconstruct the manufacturing sector that was analyzed in Stanley *et al*.^3^.

We analyzed the stability of the power law by using 5-year pooled regressions. For a given starting period, we pooled the yearly growth rates and initial sizes for all companies in that sector or industry for five subsequent years. This step is repeated by shifting the starting and end years by one. The data was pooled for five years as the year-to-year plots were noisy for smaller industries.

## Supplementary information

Supplementary Info

## References

[1] Serino, C. A., Tiampo, K. & Klein., W. New approach to Gutenberg-Richter scaling. *Phys. Rev. Lett*. **106.10** (2011). 10.1103/PhysRevLett.106.108501 21469839

[2] Gabaix X (2016). Power laws in economics: An introduction. J. Econ. Perspect..

[3] Stanley MHR (1996). Scaling behaviour in the growth of companies. Nat..

[4] Nunes Amaral, L. A. *et al*. Scaling behavior in economics: I. empirical results for company growth. *J. Phys. I Fr. cond-s* 621–633 (1997).

[5] Buldyrev SV (1997). Scaling behavior in economics: II. modeling of company growth. J. Phys. I Fr..

[6] Stanley HE, Amaral LAN, Gopikrishnan P, Plerou V, Salinger MA (2002). Application of computational statistical physics to scale invariance and universality in economic phenomena. Comput. Phys. Commun..

[7] Riccaboni M, Pammolli F, Buldyrev SV, Ponta L, Stanley HE (2008). The size variance relationship of business firm growth rates. Proc. Natl. Acad. Sci. USA.

[8] Newman M (2005). Power laws, Pareto distributions and Zipf’s law. Contemp. Phys..

[9] Reed, W. J. The Pareto, Zipf and other power laws. *Econ. Lett*. **74** (2001).

[10] Plerou V, Stanley HE, Gabaix X, Gopikrishnan P (2004). On the origin of power-law fluctuations in stock prices. Quant. Finance.

[11] Gabaix X, Gopikrishnan P, Plerou V, Stanley HE (2003). A theory of power-law distributions in financial market fluctuations. Nat..

[12] Gabaix X (2009). Power laws in economics and finance. Annu. Rev. Econ..

[13] Amaral LAN, Buldyrev SV, Havlin S, Salinger MA, Stanley HE (1998). Power law scaling for a system of interacting units with complex internal structure. Phys. Rev. Lett..

[14] Amaral LAN, Gopikrishnan P, Plerou V, Stanley HE (2001). A model for the growth dynamics of economic organizations. Phys. A.

[15] Lee Y, Amaral LAN, Canning D, Meyer M, Stanley HE (1998). Universal features in the growth dynamics of complex organizations. Phys. Rev. Lett..

[16] Buldyrev SV, Salinger MA, Stanley HE (2016). A statistical physics implementation of Coase’s theory of the firm. Res. Econ..

[17] Canning D, Amaral L, Lee Y, Meyer M, Stanley H (1998). Scaling the volatility of GDP growth rates. Econ. Lett..

[18] Bauke H (2007). Parameter estimation for power-law distributions by maximum likelihood methods. The Eur. Phys. J. B.

[19] Clauset A, Shalizi CR, Newman MEJ (2009). Power-Law Distributions in Empirical Data. SIAM Rev..

[20] Bottazzi G, Secchi A (2006). Explaining the distribution of firm growth rates. RAND J. Econ..

[21] Di Giovanni J, Levchenko AA, Ranciere R (2011). Power laws in firm size and openness to trade: Measurement and implications. J. Int. Econ..

[22] Fu, D., Buldyrev, S. V., Salinger, M. A. & Stanley, H. E. Percolation model for growth rates of aggregates and its application for business firm growth. *Phys. Rev. E* **76** (2006). 10.1103/PhysRevE.74.036118 17025719

