# Supplementary material for: Universal fluctuations in growth dynamics of economic systems
Source: Sci Rep. 2019 Jan 24;9:713. doi: 10.1038/s41598-018-38088-z (PMC6345894; doi:10.1038/s41598-018-38088-z)
Supplement: Supplementary file 1 — Supplementary Info [file 41598_2018_38088_MOESM1_ESM.pdf]

Universal fluctuations in growth dynamics of economic systems

Nathan C. Frey,[1] Sakib Matin,[1] H. Eugene Stanley,[1,2] and Michael Salinger[3]

[1] *Department of Physics, Boston University, Boston, MA 02215, USA*

[2] *Center for Polymer Studies, Boston University, Boston, MA 02215, USA*

[3] *Department of Markets, Public Policy and Law, Questrom School of Business, Boston University, Boston, MA 02215, USA*

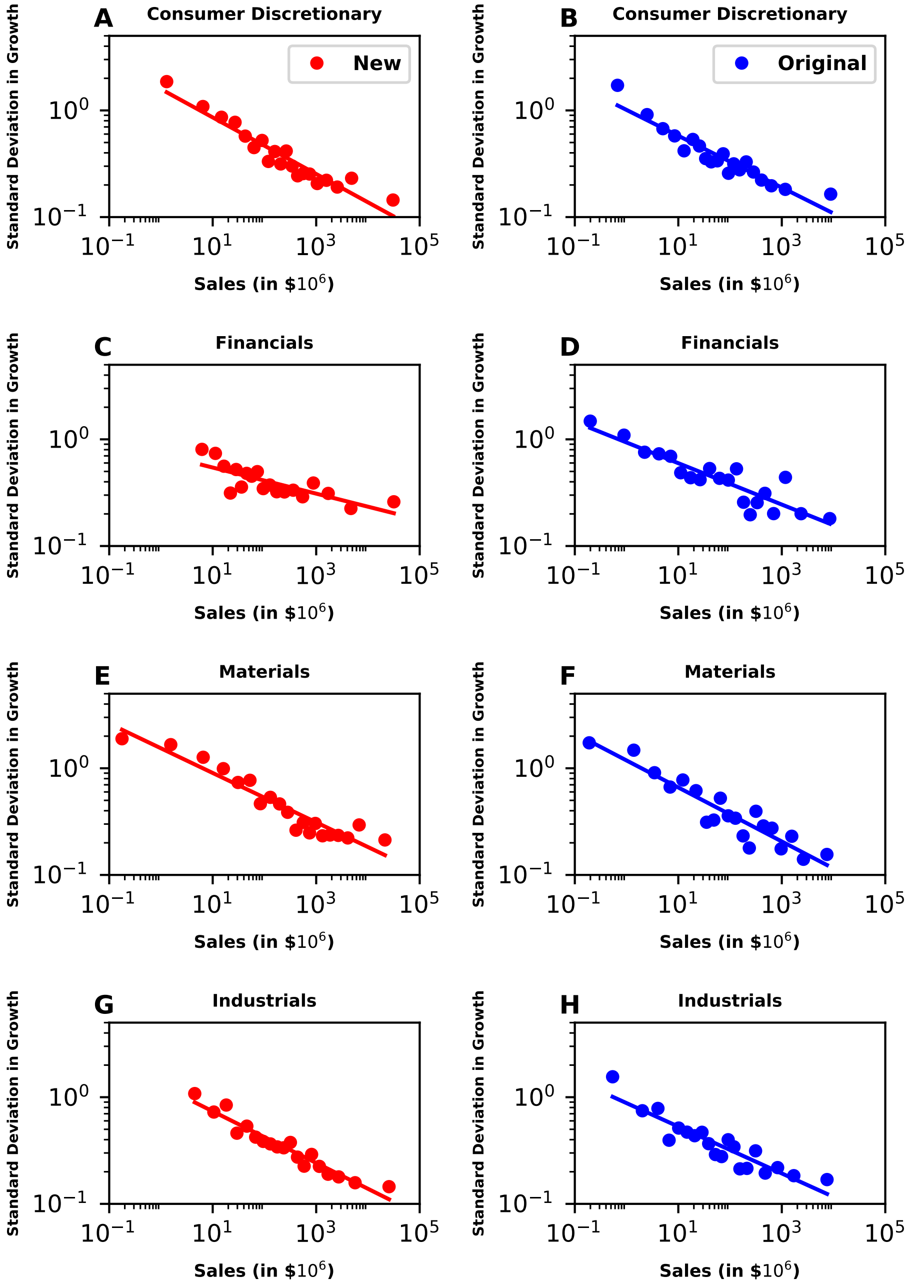

FIG. S1: Scaling of fluctuations of growth against sales for ‘Consumer Discretionary,’ ‘Financials,’ ‘Materials,’ and ‘Industrials’ in the ‘New’ (left) and ‘Original’ (right) time periods. The stability of the exponent in both time frames is strong evidence of universality.

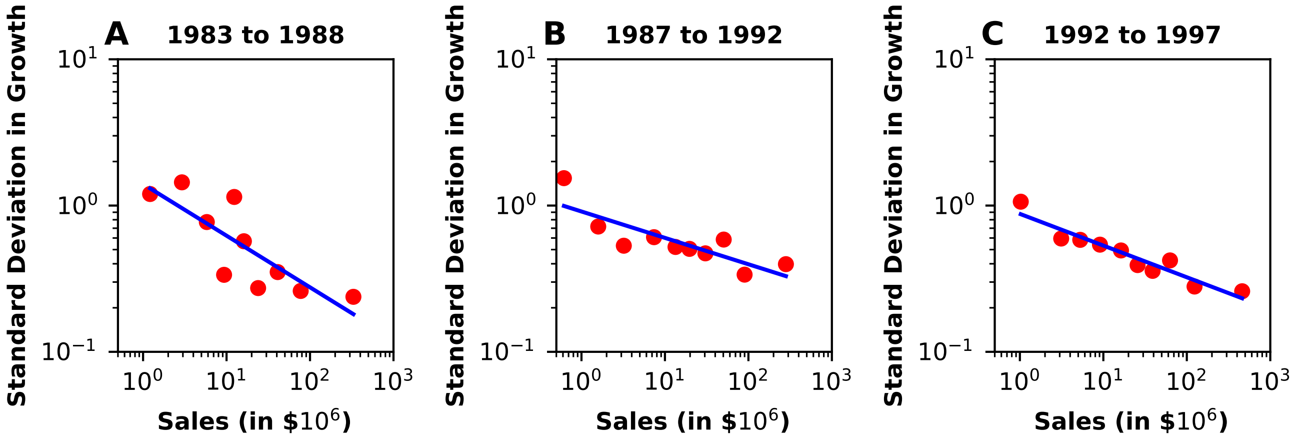

FIG. S2: Scaling of **Software** Industry at 3 distinct times showing self-organization of a power law.

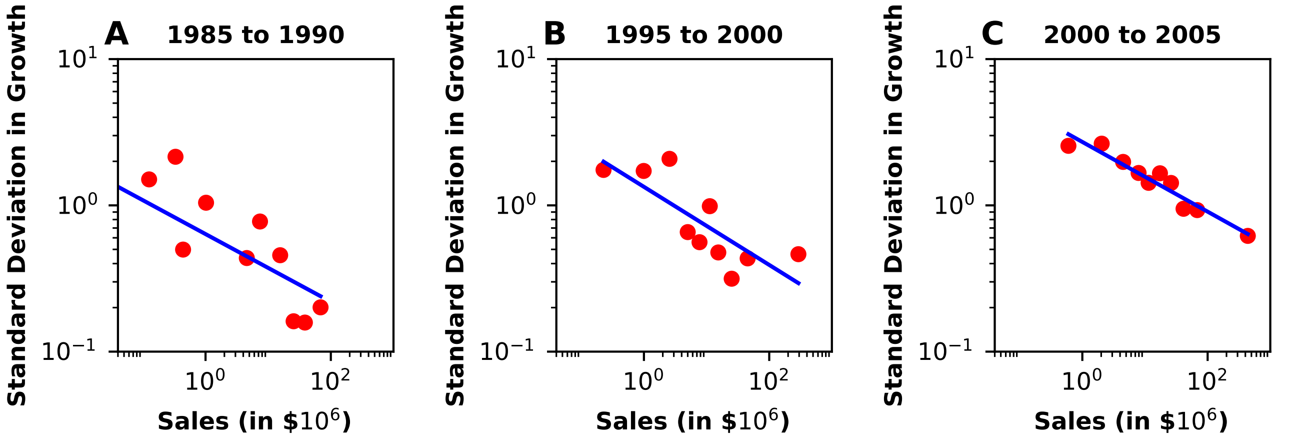

FIG. S3: Scaling of **Internet Software & Services** at 3 distinct times showing self-organization of a power law.

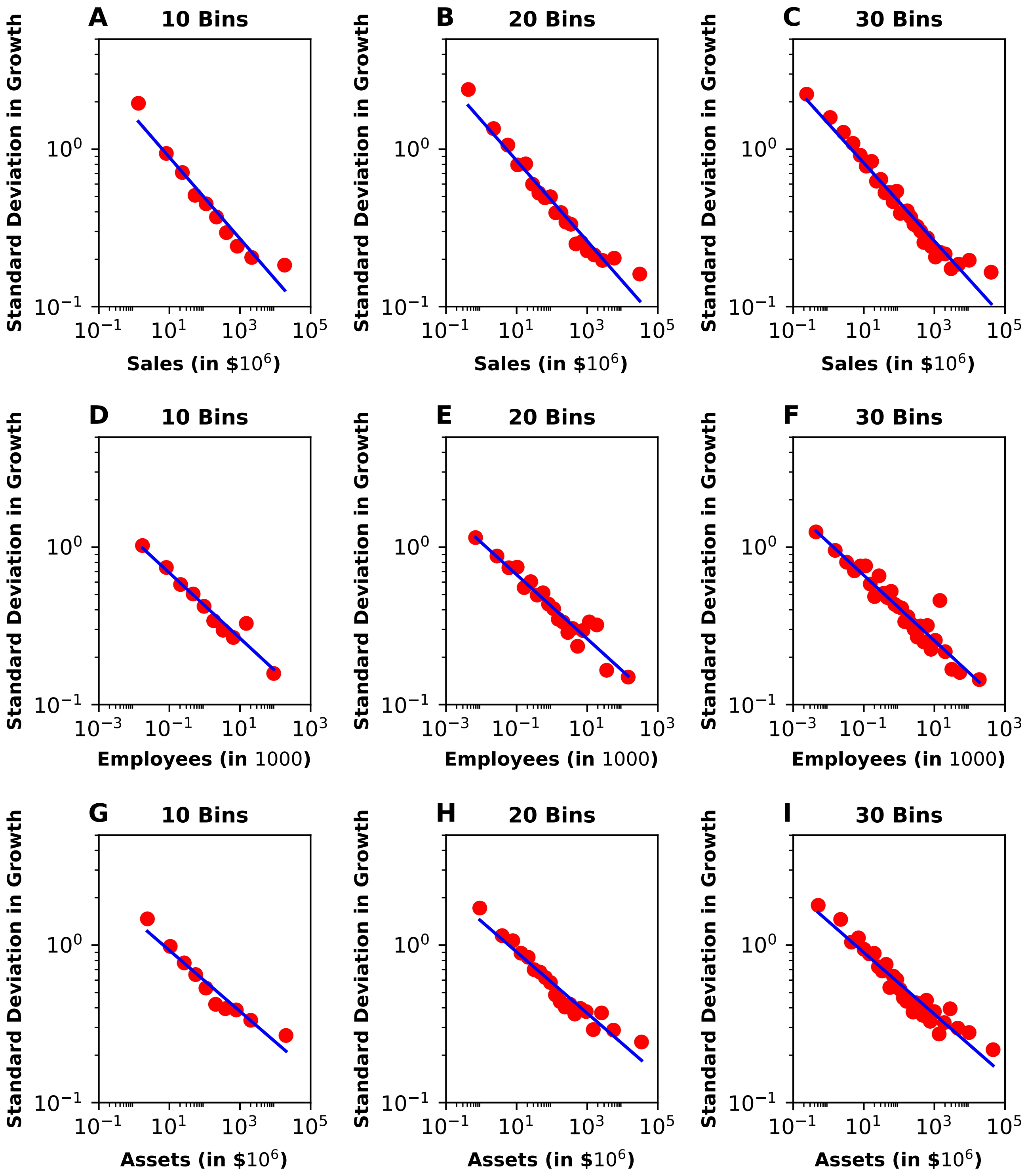

FIG. S4: Scaling of Manufacturing sector in the 'New' time period with sales, employees, and assets as measures of growth, plotted with 10, 20, and 30 bins. The value of the scaling exponent is the same (within error) regardless of the number of bins.

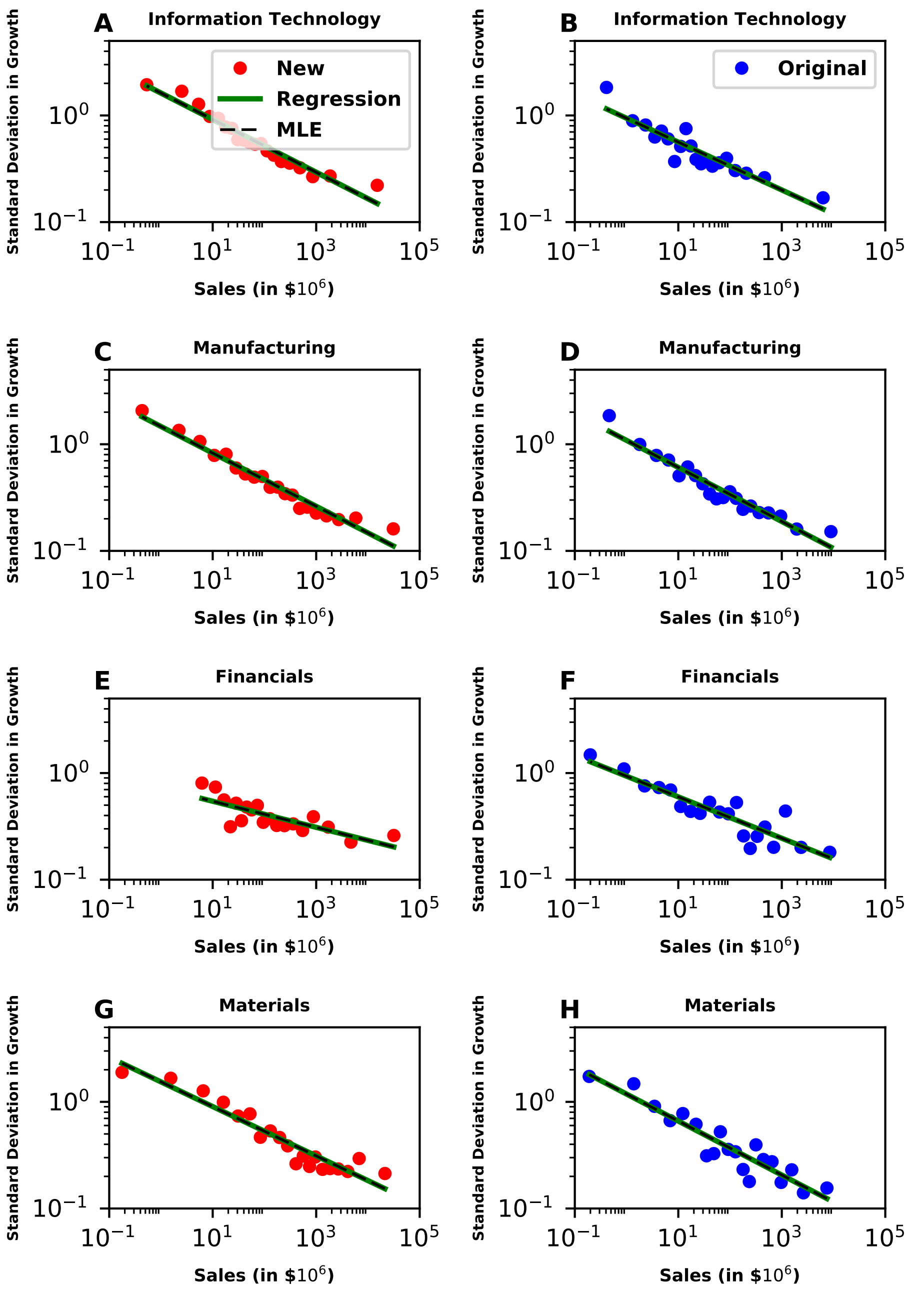

FIG. S5: Scaling exponents of fluctuations of growth against sales for 'Information Technology', 'Manufacturing', 'Financials', and 'Materials' in the 'New' (left) and 'Original' (right) time periods showing strong agreement between maximum likelihood estimation (green lines) and ordinary least squares (black dashed lines) regression.

Name	Slope	Intercept	RSqr	Std-Err	Number	Slope (MLE)	Std-Err (MLE)
Manufacturing	0.25	0.08	0.94	0.015	25527	0.25	0.014
Energy	0.19	-0.11	0.94	0.012	3413	0.19	0.011
Materials	0.25	0.17	0.88	0.022	3807	0.25	0.021
Industrials	0.22	-0.13	0.85	0.022	9372	0.22	0.021
Consumer Discretionary	0.24	0.01	0.90	0.020	10505	0.24	0.019
Consumer Staples	0.24	-0.16	0.73	0.033	2650	0.24	0.030
Health Care	0.25	0.01	0.85	0.025	3180	0.25	0.024
Financials	0.20	-0.06	0.82	0.021	5728	0.20	0.020
Information Technology	0.22	-0.06	0.85	0.022	5533	0.23	0.021
Telecommunication	0.29	0.20	0.66	0.050	1182	0.29	0.046
Utilities	0.24	-0.29	0.68	0.039	3086	0.24	0.037

TABLE S1: Summary of scaling of growth rates for all sectors classified under GICS during ‘Original’ Time. The fluctuations of the growth rates as a function of the initial size decays as a power law for all sectors except Utilities and Telecommunication.

Name	Slope	Intercept	RSqr	Std-Err	Number	Slope (MLE)	Std-Err (MLE)
Manufacturing	0.26	0.42	0.96	0.013	40908	0.26	0.012
Energy	0.19	0.47	0.94	0.012	6792	0.19	0.011
Materials	0.23	0.44	0.91	0.017	5955	0.23	0.016
Industrials	0.25	0.32	0.95	0.014	11923	0.25	0.013
Consumer Discretionary	0.27	0.47	0.92	0.019	13989	0.27	0.017
Consumer Staples	0.22	0.06	0.85	0.021	4094	0.22	0.020
Health Care	0.25	0.40	0.96	0.012	10081	0.25	0.011
Financials	0.12	-0.33	0.62	0.023	16955	0.12	0.022
Information Technology	0.25	0.48	0.94	0.014	16097	0.25	0.013
Telecommunications	0.26	0.83	0.91	0.019	2087	0.26	0.018
Utilities	0.11	-0.60	0.19	0.051	3318	0.11	0.048

TABLE S2: Summary of scaling of growth rates for all sectors classified under GICS during 'New' Time. As a function of the initial size, the fluctuations in the growth rates decrease as a power law for all sectors except Utilities and Financials.

Measure	Bins	Slope	Intercept	RSqr	Std-Err
Assets	10	0.19	0.37	0.94	0.018
Assets	20	0.20	0.35	0.94	0.012
Assets	30	0.20	0.35	0.93	0.010
Employees	10	0.21	0.85	0.95	0.017
Employees	20	0.20	0.87	0.93	0.013
Employees	30	0.21	0.89	0.91	0.012
Sales	10	0.26	0.48	0.94	0.022
Sales	20	0.26	0.42	0.96	0.013
Sales	30	0.25	0.37	0.96	0.010

TABLE S3: Summary of scaling of growth rates for the Manufacturing sector classified under GICS during ‘Original’ Time, for different measures of growth and using different number of bins.

Name	Gamma	p-Value
Manufacturing	0.0175	0.246
Energy	0.0079	0.184
Materials	0.0142	0.845
Industrials	0.0151	0.778
Consumer Discretionary	0.0232	0.120
Consumer Staples	0.0318	0.549
Health Care	0.0010	0.978
Financials	0.0089	0.912
Information Technology	0.0184	0.520
Telecommunications	0.0291	0.856
Utilities	0.1090	0.103

TABLE S4: Log-likelihood ratio test for Original time. The null hypothesis is the nonlinear term (γ) is zero. At 0.95 confidence interval we are unable to reject the null for all sectors.

Name	Gamma	p-Value
Manufacturing	0.0203	0.057
Energy	0.0111	0.190
Materials	0.0261	0.362
Industrials	0.0135	0.111
Consumer Discretionary	0.0267	0.025
Consumer Staples	0.0186	0.244
Health Care	0.0142	0.395
Financials	0.0136	0.074
Information Technology	0.0189	0.057
Telecommunication	0.0105	0.878
Utilities	0.0595	0.109

TABLE S5: Log-likelihood ratio test for New time. The null hypothesis is that the nonlinear term is zero. At 0.95 confidence interval we are unable to reject the null for all sectors except Consumer Discretionary.
